# Noninvasive imaging methods for evaluating cardiovascular involvement in patients with rheumatoid arthritis before and after anti-TNF drug treatment

**DOI:** 10.2144/fsoa-2018-0108

**Published:** 2019-06-13

**Authors:** Fabiola Atzeni, Luigi Gianturco, Laura Boccassini, Piercarlo Sarzi-Puttini, Gianluca Bonitta, Maurizio Turiel

**Affiliations:** 1 Rheumatology Unit, Department of Clinical and Experimental Medicine, University of Messina, Messina, Italy; 2 Cardiology Unit, Beato Matteo Hospital, GSD Hospitals, Vigevano, Pavia, Italy; 3 Rheumatology Unit, ASST Fatebenefratelli-Sacco, University of Milan, Italy; 4 IRCCS Orthopedic Institute Galeazzi, Milan, Italy

**Keywords:** cardiovascular disease, cardiovascular risk, carotid ultrasound, disease activity, echocardiography, myocardial alterations, noninvasive methods, rheumatoid arthritis, speckle tracking echocardiography, ventricular alterations

## Abstract

**Aim:**

To use 2D speckle-tracking echocardiography, and conventional and tissue Doppler echocardiography to detect subclinical left ventricular myocardial dysfunction in patients with rheumatoid arthritis (RA).

**Methods:**

Thirty RA outpatients were assessed before and after 18 months of treatment with anti-TNF drugs, along with 30 healthy controls. Cardiovascular risk was assessed by means of ultrasound carotid assessment and comprehensive echocardiographic evaluation (conventional and speckle-tracking calculation).

**Results:**

The speckle-tracking analyses were significantly different between the two groups, with global longitudinal strain deformation in the apical four-chamber view being significantly lower in the RA patients (median: 18.78%, interquartile range [IQR]: 15.80–20.82% vs 20.16%, IQR: 19.03–21.89%; [p < 0.05]). After 18 months of biological treatment, global longitudinal strain showed a significant improvement (18.78%, IQR: 15.80–20.82 vs 19.24%, IQR: 18.23–19.98; [p < 0.01]), such as for DAS28 (4.80, IQR: 4.65–5.22 vs 2.78; IQR: 2.52–2.99; [p < 0.01]).

**Conclusion:**

Speckle-tracking echocardiography showed that left ventricular myocardial longitudinal strain was impaired in the RA patients.

## Lay abstract

The different structures of the heart may be affected by rheumatoid arthritis (RA)-related inflammation, with the most frequent lesion being conduction defects, followed by pericarditis, cardiomyopathy and valve disease. We demonstrated, in a study involving 30 RA patients and using speckle-tracking echocardiography, that left ventricular myocardial longitudinal strain was impaired in the RA patients, in the absence of any clinical or other echocardiographic evidence of cardiovascular disease. The results suggest that inflammation is associated with myocardial alteration, as it returns to healthy controls levels upon therapy with anti-TNF drugs.

Mortality due to cardiovascular (CV) disease seems to be increased in patients with rheumatoid arthritis (RA), but there are few data concerning CV morbidity. The prevalence of ischemic heart disease is more than twice as high as in controls [1], and RA is also associated with signs of nonatherosclerotic CV disease. The different structures of the heart may be affected by RA-related inflammation, with the most frequent lesions being conduction defects, followed by pericarditis, cardiomyopathy and valve disease [2]. As accelerated atherosclerosis and signs of nonatherosclerotic CV disease both need to be detected earlier in order to protect patients against events such as myocardial infarction and malignant arrhythmias [3], researchers have been investigating innovative means of rapidly revealing CV injury [4]. These include relatively cheap ultrasonography techniques that are more reproducible than MRI, which, although it has certainly improved our knowledge and broadened our horizons, can hardly be considered for routine use. Cardiac echography is therefore more important in clinical practice [5], and the continuing introduction of new probes, machines and software has made it an increasingly comprehensive diagnostic of studying the heart. The integration of cardiac and vascular carotid ultrasonography allows the detection of atherosclerosis, and the relatively new technique of speckle-tracking echocardiography (STE) is a highly feasible and very useful means of studying cardiac mechanics and analyzing the left ventricle (LV) [6,7].

On the basis of these considerations, the aim of this study was to use 2D STE as well as conventional and tissue Doppler echocardiography to detect subclinical LV myocardial dysfunction in patients with RA.

## Methods

The study involved 30 consecutive outpatients fulfilling the 2010 America College of Rheumatology (ACR) criteria for RA [8], and 30 healthy controls matched in terms of age, gender and other anthropometric characteristics. None of the participants had smoked cigarettes during the previous 10 years.

Their socio-demographic, anthropometric and clinical data, traditional CV risk factors, comorbidities and current medications were determined by means of a standardized clinical interview. Functional impairment and disease activity were assessed using the 28-joint disease activity score (DAS28) [9] and the health assessment questionnaire [10].

The RA-related laboratory variables of erythrocyte sedimentation rate and C-reactive protein levels were measured using routine methods. Total serum cholesterol, triglyceride and high-density lipoprotein cholesterol levels were measured using an autoanalyzer, and low-density lipoprotein cholesterol levels were calculated using Friedewald’s formula.

All of the RA patients were evaluated at baseline and after 18 months of anti-TNF treatment.

The study protocol was approved by our local Ethics Committees and conduced in accordance with the Declaration of Helsinki. All of the participants gave their informed consent before undergoing any study procedure.

### Cardiovascular assessment

The patients’ CV risk profiles were determined by means of standard electrocardiography, conventional echocardiography, carotid ultrasonography with the measurement of pulse wave velocity (PWV). Arterial blood pressure and the electrocardiography findings were evaluated using standard procedures [11].

### Standard echocardiography

Transthoracic echocardiography was carried out using an iE33 ultrasound unit (Philips Medical Systems, MA, USA) equipped with a 1–2 MHz (S5) transducer and a 3–8 MHz broadband high-frequency transthoracic transducer (S8) with second harmonics. LV diameter and wall thickness were measured using targeted 2D M-mode echocardiographic traces as recommended by the American Society of Echocardiography [12]. LV wall motion was calculated by dividing the LV into 16 segments (graded 1 = normal, 2 = hypokinetic, 3 = akinetic or 4 = dyskinetic) and then dividing the sum of these scores by the number of visualized segments to obtain an overall wall motion index score [13].

LV mass was calculated using Devereux’s formula [14], and the Doppler indices of LV diastolic function were obtained using standard techniques [15].

### Speckle-tracking analysis

The speckle-tracking analysis was made offline using QLAB 9.0 software (Philips Medical Systems). The 2D images were obtained using the apical four-chamber view at a frame rate of 70–80 frames/s, and three cardiac cycles were stored in cine-loop format in order to determine end-systolic LV longitudinal strain (ϵ). The endocardial border traced on an end-diastolic frame was automatically tracked, and the tracking was verified in real time and corrected by adjusting the region of interest or manually correcting the border. End systole was considered as corresponding to aortic valve closure as determined by pulsed Doppler. The software represents myocardial deformation in the form of time-strain graphs that allow the different phases of the cardiac cycle to be identified: maximal longitudinal myocardial shortening during contraction is reached when the negative wave peaks during systole at the time of aortic valve closure; the strain values during diastole gradually return toward the original length.

The time-strain curves were analyzed by two independent observers who were blinded to the clinical data. Interobserver variability was <5%, as established using the Bland–Altman method to compare the two observers’ measurements of ten randomly selected subjects [16].

### Carotid artery ultrasonography & stiffness measurements

Carotid artery ultrasonography was carried out using a My Lab 60 (Esaote, Florence, Italy) equipped with a 2–9 MHz LA532E linear array transducer and RF-QIMT and RF-QAS software complying with the Mannheim Consensus [17]. The intima-media thickness of the common carotid artery was measured 1 cm distally of the carotid bifurcation in the posterior wall of the right and left carotid arteries, and defined as the distance between the leading edges of the lumen interfaces and the media/adventitia interface of the far wall [18,19]. The average of three measurements was recorded.

Arterial stiffness was assessed by examining the right and left common carotid arteries about 1 cm proximally of the bulb region and using the time-related pressure waveforms obtained from the systolic and diastolic changes in arterial diameter after calibrating blood pressure in the right upper arm using a cuff manometer. PWV is a basic parameter of vascular stiffness, and is usually assessed on the basis of two-point measurements and how long it takes for the wave to travel a known distance; however, it can also be assessed on the basis of a one-point measurement. We used the radio frequency processing of linear amplitude and phase data, which automatically, highly accurately and noninvasively provides real-time measurements of the changes in vessel wall diameter (distension) and wall thickness. These are then be used to calculate the stiffness biomarkers of local PWV and quality intima-media thickness (QIMT), which are normalized to local blood pressure by re-scaling the local distension waveform to brachial pressure [20].

### Statistical analysis

Continuous variables are expressed as mean and standard deviations, or median and interquartile ranges (IQRs) depending on their skewness, and were compared using Mann–Whitney test Wilcoxon, signed rank test or t-test, Chi-square test as appropriate; categorical variables are expressed as percentages. The data were analyzed using the free statistical R software package, and a p-value of < 0.05 was considered statistically significant.

## Results

Thirty outpatient RA patients (14 males and 16 females; mean age 54.63 ± 9.36 years; median disease duration 2 years) and 30 healthy controls with comparable characteristics were included in the study (Table 1).

**Table 1.** Baseline characteristics of the study population.

Parameters	RA patients (n = 30)	Healthy controls (n = 30)	p-value
Number of females (%)	16 (53%)	15 (50%)	0.999
Age (years)	54.63 ± 9.36	52.5 ± 10.6	0.999
BMI (kg/m^2^)	20.0 ± 1.8	20.0 ± 2.0	0.4127
Nonsmokers	30 (100%)	29 (97%)	0.999
Hypertension			
Systolic blood pressure (mmHg)	134.1 ± 12.5	136.4 ± 22.1	0.622
Diastolic blood pressure (mmHg)	80.1 ± 8.2	80.8 ± 9.4	0.759
Disease duration (months)	24		
Rheumatoid factor positive (%)	25 (83%)		
Anti-CCP positive (%)	23 (77%)		
DAS28 (score)	4.76 ± 0.82		
Total cholesterol (mg/dl)	178.3 ± 21.3	180.6 ± 20.8	0.6737
HDL cholesterol (mg/dl)	52.7 ± 12	57.5 ± 13.1	0.142
LDL cholesterol (mg/dl)	105.2 ± 14.5	101.0 ± 15.7	0.286
Triglycerides (mg/dl)	112.7 ± 14.6	119.3 ± 15.7	0.100
Glycemia (mg/dl)	87.6 ± 9.8	85.0 ± 15.0	0.413
CRP (mg/dl)	14.04 ± 15.3	0.3 ± 0.2	< 0.001
ESR (mm/h)	38.2 ± 22.5	7.9 ± 5.6	< 0.001

Mean values ± SD unless otherwise indicated.

*p < 0.01.

BMI: Body mass index; CCP: Cyclic citrullinated peptide antibody; CRP: C-reactive protein; DAS28: Disease activity score; ESR: Erythrocyte sedimentation rate; HDL: High-density lipoprotein; LDL: Low-density lipoprotein; RA: Rheumatoid arthritis.

27 (90%) of the RA patients were being treated with methotrexate at baseline; their mean DAS28 was 3.9 ± 2.4, and their mean health assessment questionnaire score was 1.0 ± 0.5. After a CV evaluation, they were treated with anti-TNF drugs in combination with methotrexate 7.5–10 mg/week; five patients also received corticosteroids 5 mg/day.

None of the patients showed any signs or symptoms of CV disease, pulmonary involvement or any other complication. The mean left ventricular ejection fraction (LVEF) and E/A ratio (marker of the function of the left ventricle) were, respectively, 58.43 ± 3.07% and 0.75 ± 0.35, and were not significantly different from those of the controls (60.45 ± 5.24% and 0.85 ± 0.29; p = 0.08 and 0.233). The results of the speckle-tracking analysis were significantly different between the two groups, with global longitudinal strain deformation in the apical four-chamber view being significantly lower in the RA patients (median 18.78%, IQR: 15.80–20.82% vs 20.16%, IQR: 19.03–21.89%; p < 0.05). In comparison with the controls, the patients’ median right and left PWV was greater (7.92 m/s; IQR: 7.14–8.60 vs 6.85 m/s; IQR: 6.41–7.88; p = 0.07; and 7.90 m/s; IQR: 6.99–8.16 vs 6.85 m/s; IQR: 6.36–7.84; p = 0.06), and their median right and left common carotid intima-media thickness (cIMT) was significantly greater (0.90 mm; IQR: 0.75–1.08 vs 0.75 mm; IQR: 0.50–0.75; p < 0.05; and 0.89 mm; IQR: 0.74–0.99 vs 0.74 mm; IQR: 0.49–0.85; p < 0.05). Furthermore, there was a significant improvement in median end-systolic LV longitudinal strain in the patients after 18 months of biological treatment (18.78%; IQR: 15.80–20.82 vs 19.24%; IQR: 18.23–19.98; p < 0.01), and a minimal reduction in arterial stiffness and cIMT parameters (p = not significant [NS]). Finally, DAS28 was also better after treatment in patients (4.80; IQR: 4.65–5.22 vs 2.78; IQR: 2.52–2.99; p < 0.01)].

## Discussion

These findings show that there were no differences in the standard echocardiographic parameters of systolic function (including the LVEF and myocardial velocity) between our RA patients and healthy controls, but the patients did show impaired LV longitudinal strain in the absence of clinical or signs of CV involvement suggesting a myocardial alteration.

The most widely used index of LV contractile function is the LVEF, but the visual component of the assessment of endocardial excursion makes it subject to a high degree of inter- and intraobserver variability. Our results are in line with these of Baktir *et al*. [7], who found statistically insignificant higher E/E’ ratios in their patients with long-lasting RA than in controls, wheareas Abdul Muizz *et al*. [21] found statistically insignificant lower E/E’ ratios in their patients. We did not evaluate the correlation between the ratios and disease duration in our population, but Di Franco *et al*. [22] found that it was close, and Montecucco *et al*. [23] have found correlations between disease duration and both 2D LV relaxation parameters and the E/A ratio in patients with RA; however, Rexhepaj *et al*. [24] did not find any correlation between Doppler tissue echocardiography findings and the duration of RA other than a weak correlation with the E/A ratio.

The value of standard echocardiography in assessing LV contractile function is limited by its angle dependence and poor spatial resolution, as well as the difficulty of analyzing deformation in one dimension [25], whereas 2D STE is more widely used because it evaluates LV systolic function more objectively and quantitatively, and does not have the same limitations [26]. As shown in our previous study [27], impaired myocardial deformation can be detected by means of STE in patients with long-standing RA even if they show normal 2D echocardiographic systolic LV function and have a normal wall motion index at rest and during echostress. The findings of this new study not only confirm that STE can detect impaired LV longitudinal/global systolic strain in RA patients despite the normal systolic parameters revealed by Doppler echocardiography, but also indicate a simultaneous carotid artery alteration. This suggests the involvement of both the myocardium and atherosclerosis, and is indicative of the greater role played by the chronic systemic inflammation that characterizes RA.

Our findings are in line with these of Baktir *et al.* [7], who showed that STE can reveal impaired myocardial deformation in RA patients before the appearance of any clinical evidence of cardiac involvement, which correlates with the clinical characteristics of RA and the markers of cardiac damage found by Benacka *et al.* [28]. This systolic dysfunction may be due to chronic systemic mechanisms as it is known that inflammatory cytokines such as TNF-α and IL-6 play a pathogenic role in LV remodeling by affecting heart contractility, inducing hypertrophy, and promoting apoptosis and fibrosis [29,30].

Various studies have shown that anti-TNF drugs act on the symptoms and signs of RA, and therefore on atherosclerosis – as is shown by albeit nonsignificant improvement in the carotid alteration and arterial stiffness of our patients [31]. Ayyildiz *et al.* [32] found that long-term anti-TNF therapy also improves LV longitudinal and radial systolic deformation and decreases left ventricular torsion in RA patients. Our findings suggest that TNF-α plays a central role as an inflammatory marker of atherosclerosis, and predicts CV events in healthy subjects, whereas long-term TNF inhibition improves LV longitudinal and radial systolic deformation, which may even normalize during the course of treatment.

The main limitation of our study is the small number of patients and the short period of follow-up; further studies of larger samples of RA patients are required in order to define more precise methods of assessing CV disease and the benefits of anti-TNF therapy. However, the strength of the study is its use of noninvasive methods that are widely used in clinical practice.

In conclusion, LV longitudinal strain is significantly impaired in RA patients, in the absence of any clinical or other echocardiographic evidence of CV disease, but improves after 18 months of anti-TNF treatment, which also leads to a minimal reduction in arterial stiffness and cIMT.

The results suggest that inflammation is associated with myocardial alteration, as it returns to healthy controls levels upon therapy with anti-TNF drugs.

## Conclusion & future perspective

On the basis of the favorable data from this study, we hope the use of this technique will become greater in the future; we expect that it could significantly change the course of early diagnosis of myocardial involvement in RA. The results cannot be applied to patients with established CVD, hypertension and diabetes, who were excluded from the current study, but because RA patients suffer from several comorbidies we hope to also extend the use of this noninvasive method to these patients. The 2D STE is a valuable and reproducible tool for detecting impairment of left and right ventricle systolic function in RA patients, even in the presence of normal ejection fraction. The degree of systolic function impairment improved during anti-TNF treatment in our study, as seen with DAS28. This raises the concern that inappropriate management of RA activity could lead to development of heart failure. Finally, we hope that prospective studies with larger numbers of patients and more long-term follow-up will be assessed in the near future in patients with RA in order to treat them early and with an appropriate therapy chosen from among those available on the market.

**Summary points**

- No differences in the standard echocardiographic parameters of systolic function (including the left ventricular ejection fraction and myocardial velocity) were observed between our rheumatoid arthritis patients and healthy controls.
- Rheumatoid arthritis patients did show impaired left ventricle longitudinal strain in the absence of clinical signs of cardiovascular involvement, suggesting a myocardial alteration.
- Anti-TNF treatment improved left ventricle longitudinal and radial systolic deformation.
- Our data suggest that inflammation is associated with myocardial alteration, as it returns to healthy controls levels upon therapy with anti-TNF drugs.
